# Patient-Reported Outcome Measures Used on Patients With Anterior Cruciate Ligament Injury

**DOI:** 10.7759/cureus.64546

**Published:** 2024-07-15

**Authors:** Apostolos D Prodromidis, Georgios C Thivaios, Anastasios Mourikis, Ioannis D Erginousakis, Vasileios S Nikolaou, John Vlamis, Efstathios Chronopoulos

**Affiliations:** 1 Orthopaedics, School of Medicine, National and Kapodistrian University of Athens, Athens, GRC; 2 Orthopaedics and Trauma, Laiko General Hospital of Athens, Athens, GRC; 3 Orthopaedics and Trauma, KAT Attica General Hospital, Athens, GRC; 4 Medicine, School of Medicine, University of Patras, Patra, GRC; 5 Orthopaedics and Trauma, Konstantopoulio General Hospital, Athens, GRC; 6 Musculoskeletal System Research Laboratory, KAT Attica General Hospital, Athens, GRC

**Keywords:** patient-reported outcome (pro), patient-reported outcome measures, knee scores, quality of life, outcome measures, proms, patient-reported outcomes, acl surgery, acl injury

## Abstract

Patient-reported knee-related rating scores and scales are widely used in reporting the clinical outcomes of anterior cruciate ligament (ACL) surgery. Understanding the psychometric properties of such measures is vital to recognizing the limitations that such measures may confer. The aim of this study was to review the available evidence as to the psychometric properties of patient-reported outcome measures (PROMs) used in ACL surgery. Eleven studies were identified, the majority being prospective cohort studies. Eight English, ACL-specific patient-reported outcome measures were identified and evaluated: Lysholm score, Tegner Activity Scale (TAS), Cincinnati score, ACL-Quality of Life (QOL) score, International Knee Documentation Committee (IKDC) Subjective Knee Form (SKF), Knee Injury and Osteoarthritis Outcome Score (KOOS)-ACL score, and ACL-Return to Sport Injury (RSI) scale. Only the Lysholm score, ACL-QOL, IKDC SKF, and ACL-RSI were evaluated for internal consistency, having an acceptable Cronbach’s α (α>0.70). Most of the scoring systems were assessed for test-retest reliability, with four of them (Lysholm score, TAS, Cincinnati score, and IKDC SKF) having acceptable intraclass correlation coefficient (ICC) values (ICC > 0.70). Criterion validity was assessed for most measures with a good correlation with the IKDC. Effect sizes and standardized response means were large for three instruments that measured responsiveness (Lysholm score, TAS, and Cincinnati score) and moderate for one (ACL-QOL). Evidence is stronger and more robust for the Lysholm score, TAS, ACL-QOL, and IKDC SKF. However, there is variation in their psychometric properties as well as the aspect of knee-related health they are assessing. Hence, none can be universally applicable to all patients with ACL injuries. Recognizing these parameters is vital when choosing which instrument to use in reporting the outcomes of ACL injury or ACL surgery studies.

## Introduction and background

Anterior cruciate ligament (ACL) injuries are increasingly common in young adults, and ACL reconstruction (ACLR) surgery for such injuries is a commonly performed procedure in sports medicine, especially in younger and more athletic patients [1]. Hence, evaluating accurately the short- and long-term outcomes following treatment for ACL injuries is essential.

Over the past few decades, there has been a significant increase in the development of patient-reported outcome measures (PROMs), with patient-based knee scoring systems and rating scales designed to assess outcomes following ACL injury and/or ACL surgery [2-4]. However, the interpretation of such outcomes is not easy, and their significant change over time may not be clinically relevant [3]. Moreover, thresholds for acceptable or good patient-reported outcome scores are not known.

Patient-reported outcome measures are tools used to assess patients’ health status, evaluating different aspects of their health status relevant to their quality of life from their perspective. Such aspects include symptoms, functionality, and physical, mental, and social health. The quality of the information obtained by these tools is closely related to the psychometric properties of those measures. The psychometric properties of outcome measures include such parameters as reliability, validity, and responsiveness [5, 6]. Reliability refers to whether a tool can consistently reproduce the same results over time; validity refers to whether an outcome or tool measures what is designed to measure; and responsiveness is how an instrument reflects and can measure changes over time [6, 7]. Collectively, all these parameters are psychometric properties and reflect the methodological quality of a tool, scale, or outcome measure [8].

The selection of an outcome assessment tool that reliably measures the outcomes following the treatment of a condition is crucial to making valid comparisons between different strategies or techniques, especially when it comes to surgery, and deciding on the best treatment for patients.

The aim of this study is to review and evaluate all available PROMs for the knee, focusing on ACL injuries.

## Review

Methods

The Cochrane methodology for systematic reviews was followed [9]. The predefined protocol was published in the International Prospective Register of Systematic Reviews (PROSPERO, CRD42024545976). A literature search of Medical Literature Analysis and Retrieval System Online (MEDLINE, EBSCOhost) and Cumulative Index to Nursing and Allied Health Literature (CINAHL, EBSCOhost) with no publication year limit was performed in May 2024. Only studies available in English were included. The search in both databases was developed by combining the following set of keywords with the Boolean operator AND: [patient reported outcomes OR functional outcome measure* OR scor*] AND [anterior cruciate ligament OR ACL] AND [injury OR tear OR rupture OR surgery]. Full texts were reviewed for relevant articles or where a decision regarding inclusion could not be made based on title and abstract. The reference lists of all selected papers were also scrutinized for any additional relevant papers not identified with the database search.

Patient-reported outcome measures designed to be administered following ACL injury in adult patients were the outcome measures of interest. Only English versions of such outcome measures were considered. General health measures (such as the 36-item Short Form Health Survey) were excluded. Measures used in heterogeneous populations with additional non-knee and non-ACL-related issues (such as general knee pain or hip or ankle problems) were excluded. Only adult patients (age ≥ 16 years) with confirmed ACL injuries were included.

Randomized controlled trials (RCTs), prospective and retrospective cohort studies, case-control studies, and cross-sectional studies were included. Only studies specifically set out to evaluate the psychometric properties of patient-reported outcomes designed for patients with ACL injuries were included. Case reports, reviews, editorials, commentaries, personal opinions, and surveys were excluded. The methodology of the studies was classified according to Mathes and Pieper [10].

Data were extracted using a standardized data extraction form and inputted onto a Microsoft Excel (Microsoft Corp., Redmond, WA) spreadsheet to record all results. The following data were extracted for each study: (i) Study characteristics: design, year, country, level of evidence, number of patients; (ii) Patient population characteristics: age, gender; (iii) PROM instruments and relevant scoring; (iv) Methods of developing and testing each instrument; (v) Psychometric properties of each instrument, including reliability, validity, and responsiveness; (vi) Risk of bias assessment data using the following tools: The Cochrane Risk of Bias Tool was used for RCTs [11] and the Newcastle-Ottawa Scale (NOS) for prospective cohort studies [12].

Definitions of the Psychometric Parameters Used

Reliability: Two measures used were internal consistency and test-retest reliability. Internal consistency measures whether different items or subscales of the same test that are designed to measure the same dimension or construct give similar scores. It can be evaluated with Cronbach’s alpha (α). In Cronbach’s α analysis, a score > 0.70 is considered acceptable, although it is influenced by the sample size [13]. Test-retest reliability refers to the variation of results over time and is the degree to which the test scores remain unchanged when measuring a stable individual characteristic on different occasions. Test-retest reliability is most commonly calculated with the intraclass correlation coefficient (ICC). The ICC ranges from 0 to one, with an acceptable range for patients within a clinical trial being ≥ 0.70 [14].

Validity: This comprised construct, content, and criterion validity. Construct validity refers to the extent to which a test/measure accurately assesses what it is designed to measure. Construct validity integrates different forms of validity (content, criterion), and it is measured with either exploratory factor analysis (EFA) or confirmatory factor analysis (EFA). Content validity is the extent to which an instrument represents all aspects of the topic or construct it is supposed to measure. Criterion validity refers to the extent to which a measure agrees with an accepted “gold standard” instrument. Because a single “gold standard” instrument is rarely accepted, researchers generally administer other similar instruments along with the instrument of interest and compare correlations.

Responsiveness: Usually, after intervention, the estimation of responsiveness is based on effect size (ES) and standardized response mean (SRM). The ES is calculated using the baseline SD, which is the average difference divided by the standard deviation of the first measurement. The ES is calculated using the pooled SD, which is the average difference divided by the pooled standard deviation of both measurements. The SRM is the average difference divided by the standard deviation of the differences between the paired measurements [7].

Results

Included Studies

**Figure 1:**
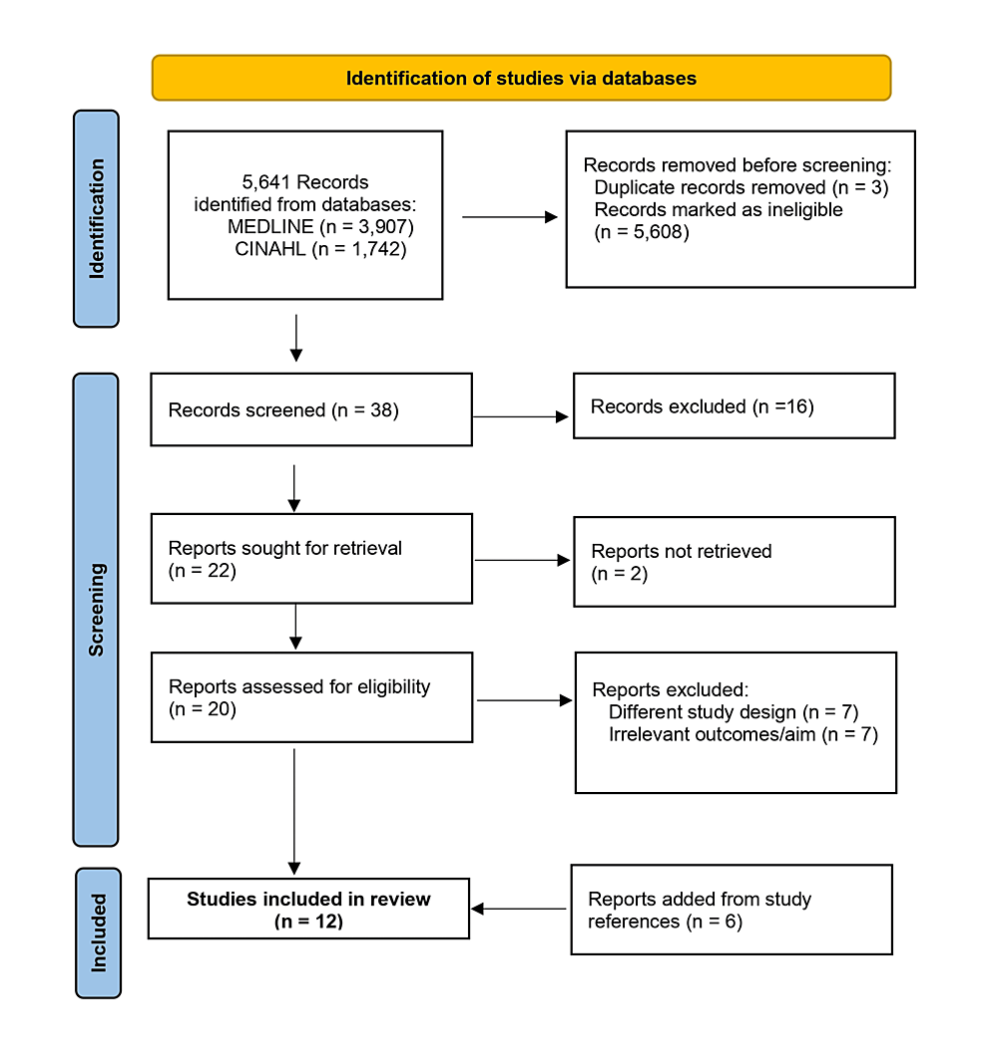
A PRISMA flow diagram of the included studies PRISMA: Preferred Reporting Items for Systematic Reviews and Meta-Analyses; MEDLINE: Medical Literature Analysis and Retrieval System Online; CINAHL: Cumulative Index to Nursing and Allied Health Literature [27]

A total of 5,641 studies were identified by title. The screening process led to the initial selection of 41 titles based on information gathered from the titles. After the removal of two duplicates and a review of 39 abstracts, 22 articles were excluded. A full-text review of the remaining 17 articles and a thorough search of their references were performed. Six of these articles and six of their references met the inclusion criteria. Therefore, 12 studies evaluating PROMs were included, and their results are presented in this review [15-26]. The process of identification of the studies is presented in a Preferred Reporting Items for Systematic Reviews and Meta-Analyses (PRISMA) flow diagram in Figure 1 [27].

Table 1 summarizes the characteristics of all 12 studies published between 1985 and 2023, with a total of 7,547 patients [15-26]. The majority of the studies were prospective cohort studies [15-18, 20, 21, 24-26], one was an RCT [22], and two were prospective case series studies [19, 23]. Most of the instruments/scores were evaluated in patients in the USA [15, 17, 18, 22], while others were in Canada [19-21], Australia [24-26], and Sweden [16, 23]. The earliest reported measure was the Lysholm score [23], and the newest was the Knee Injury and Osteoarthritis Outcome Score (KOOS)-ACL score [20]. Five studies were the original studies, which introduced the relevant instruments [17, 21-23, 25], two studies modifying validated instruments to shorter versions [18, 20, 24], and four studies comparing or evaluating the instruments further [15, 16, 19, 26].

**Table 1:** Characteristics of all the studies included in the review PRO: patient-reported outcome; M: male; F: female; ACL: anterior cruciate ligament; TAS: Tegner-Activity Scale; NR: not reported; USA: United States of America; RCT: randomized controlled trial; QOL: quality of life; IKDC: International Knee Documentation Committee; SKF: Subjective Knee Form; KOOS: Knee Injury and Osteoarthritis Outcome Score; RSI: Return to Sport after Injury

Author (year)	Design (level of evidence, country)	PRO measure	Number of patients	Sex (M:F)	Age, years mean ± SD (range)	Original study
Barber-Westin et al., (1999) [15]	Prospective cohort (II, USA)	Cincinnati score	350	227:123	29	No
Briggs et al., (2009) [16]	Prospective cohort (II, Sweden/USA)	Lysholm score, TAS	1783	1034:749	37 (18-77)	No
Irrgang et al., (2001) [17]	Prospective cohort (II, USA/France/Japan)	IKDC SKF	533	252:227 (NR: 54)	37.5±16.2	Yes
Jacobs et al., (2018) [18]	Prospective cohort (II, USA)	KOOS_ global _(short form)	1904	NR	NR	No
Lafave et al., (2017) [19]	Prospective case series (III, Canada)	ACL-QOL score	579	302:277	27.81±6.9	No
Marmura et al., (2023) [20]	Prospective cohort (II, Canada)	KOOS-ACL score	606	294:312	19±3	No
Mohtadi et al., (1998) [21]	Prospective cohort (II, Canada)	ACL-QOL score	79	NR	(16-45)	Yes
Noyes et al., (1991) [22] [22]	RCT (I, USA)	Cincinnati score	50	NR	NR	Yes
Tegner et al., (1985) [23]	Prospective case-series (NR, Sweden)	Lysholm score, TAS	76	55:21	NR	Yes
Webster et al., (2018) [24]	Prospective cohort (II, Australia)	ACL-RSI (short version)	535	347:188	26.8±9	No
Webster et al., (2008) [25]	Prospective cohort (II, Australia)	ACL-RSI scale	220	124:96	29.2±9.7 (16-54)	Yes
Webster et al., (2022) [26]	Prospective cohort (II, Australia)	KOOS_ global _(short form), IKDC SKF	832	489:343	30±9.4	No

*Content of* *Outcome Measures*

The outcome measures identified and their content are summarised in Table 2.

**Table 2:** Outcome measures TAS: Tegner-Activity Scale; ACL: anterior cruciate ligament; QOL: quality of life; IKDC: International Knee Documentation Committee; SKF: Subjective Knee Form; KOOS: Knee Injury and Osteoarthritis Outcome Score; RSI: Return to Sport after Injury; VAS: Visual Analogue Scale; MODEMS: Musculoskeletal Outcomes Data Evaluation and Management Scale; JR: joint replacement

Outcome measure	Item sources	Domains/Scale/Scoring	Scale/Scoring (points)
Cincinnati score [15, 22]	Two occupational rating systems	6 domains: pain, catching/locking, work activity, exercise program, follow-up progress, patient grade	Scale: 120-420
Lysholm score [16, 23]	Marshall scoring scale	8 domains: limb, support, locking, instability, pain, swelling, stair climbing, squatting	Scale: 0-100
TAS [16, 23]	Lysholm score	1 domain: level of activity	Scale: 0-10 level, scores 5-10: sports
IKDC SKF [17, 26]	Previous version, MODEMS instrument [28], Knee outcome survey [29]	3 domains: 9 questions, symptoms, sports activities, function	Scale: 0-100
KOOS _global _(short form) [18, 26]	Original KOOS JR [30] Original KOOS [4]	4 domains: 11 questions, stiffness, pain, function (daily living), quality of life	Scale: 0-100
ACL-QOL score [19, 21]	Expert panel (consensus method)	5 domains: 32 items/31 questions, symptoms, work-related concerns, recreational activities + sports participation, lifestyle, social and emotional	10 cm VAS format
KOOS-ACL score [20]	Original KOOS [4]	2 domains: 12 questions function, sports	Scale: 0-100
ACL-RSI scale (short version) [24]	ACL-QOL [21] ACL-RSI [25]	3 domains: 6 items/questions emotions, confidence in performance, risk appraisal	Score: 0-100
ACL-RSI scale [25]	ACL-QOL [21]	3 domains: 12 items/questions emotions, confidence in performance, risk appraisal	10 cm VAS format

All were ACL-injury-specific measures and included the Lysholm score [23], the Tegner Activity Scale (TAS) [23], the Cincinnati score [22], the ACL-Quality of Life (QOL) score [21], the KOOS global (short form) [18], the KOOS-ACL score [20], the ACL-Return to Sport after Injury (RSI) scale [25], and the short version of the ACL-RSI scale [24]. One knee-specific measure, the International Knee Documentation Committee (IKDC) score, was modified to be ACL-injury-specific, called the IKDC Subjective Knee Form (SKF) [17].

Five outcome measures were designed and administered following an ACL injury/tear not necessarily treated with surgery [17, 21-23]. However, in later studies evaluating the psychometric properties of these measures/questionnaires, they were administered only to patients who had ACL reconstruction surgery [15, 16, 19]. The rest of the four outcome measures were administered before and after ACL reconstruction surgery [18, 20, 24, 25].

There was an overlap in some health dimensions evaluated among measures, including symptoms, pain, locking, function, and sports. However, even these dimensions/domains were evaluated with different approaches (questions and possible answers). Moreover, some different dimensions were evaluated only in one or two specific measures (like stiffness, confidence in performance, and risk appraisal).

The majority of the measures used ordinal scales, apart from the ACL-QOL score [21] and the ACL-RSI score [25], which used the Visual Analogue Scale (VAS) format. However, even these two measures converted the results to an ordinal scale from 0 to 100.

Psychometric Properties

The psychometric properties of the outcome measures are presented in Table 3.

**Table 3:** Psychometric properties of all outcome measures TAS: Tegner Activity Scale; ACL: anterior cruciate ligament; QOL: quality of life; IKDC: International Knee Documentation Committee; SF-12: 12-Item Short-Form Health Survey; PC: physical component; MC: mental component; SKF: Subjective Knee Form; KOOS: Knee Injury and Osteoarthritis Outcome Score; RSI: Return to Sport after Injury; α: Cronbach’s alpha; ICC: intraclass correlation coefficient; SEM: standard error of mean; 95%CI: 95% confidence interval; r: Pearson's correlation coefficient; rho: Spearman's correlation coefficient; FCE: floor and ceiling effects; EFA: exploratory factor analysis; CFA: confirmatory factor analysis; NA: not applicable; NR: not reported; SRM: standardised response mean

Outcome measure	Internal consistency	Test-retest reliability	Content validity	Criterion validity	Factor structure	Responsiveness effect size	Responsiveness SRM
Cincinnati score [15, 22]	NR	ICC>0.70 (range: 0.756-0.98)	NR	FCE: no floor effects, 9% ceiling effects	NR	7 of 8 categories: 1.18 – 3.49 (large)	7 of 8 categories: 1.07 – 2.48 (large)
Lysholm score [16, 23]	α=0.72	ICC: 0.94 (95%CI: 0.88-0.96)	Overall mean: 57±21 FCE < 30%	IKDC: r = 0.78, p<0.001. SF-12 PC: r =0.43, p≤0.001 SF-12 MC: r = 0.07	EFA one factor	1.1 (large)	0.93 (large)
TAS [16, 23]	NR	ICC=0.82 (95%CI: 0.66-0.89)	Overall mean: 4 FCE < 30%	IKDC r = 0.22, p<0.001, SF-12 PC, r = 0.2, p<0.05, SF-12, MC: r = -0.01	ΝΑ	1.0 (large)	1.0 (large)
IKDC SKF [17, 26]	α=0.92	ICC=0.94 (95%CI: 0.88-0.97)	No floor effects, 0.2% ceiling effect	IKDC physical: r=0.66 IKDC mental: r=0.16	EFA one factor	NR	NR
KOOS _global _(short form) [18, 26]	NR	NR	No FCE	IKDC: rho=0.9, p<0.001	NR	NR	NR
ACL-QOL score [19, 21]	α=0.98	ICC=0.60 SEM: 6.16, CI(95%): ±12.1	FCE 1%	NR	NR	0.61 (moderate)	NR
KOOS-ACL score [20]	α=0.79-0.9	NR	>15% ceiling effects, No floor effects	IKDC: rho > 0.70	EFA-CFA two factor	NR	NR
TAS [16, 23]	NR	ICC=0.82 (95%CI: 0.66-0.89)	Overall mean: 4 FCE < 30%	IKDC r = 0.22, p<0.001, SF-12 PC r = 0.2, p<0.05, SF-12, MC: r = -0.01	ΝΑ	1.0 (large)	1.0 (large)
ACL-RSI scale [25]	α=0.96	ICC: 0.69 Range: 0.49-0.83	NR	NR	EFA one factor	NR	NR

Reliability: Lysholm score, ACL-QOL, IKDC SKF, and ACL-RSI (both original and short version) were evaluated for internal consistency, and they had an acceptable Cronbach’s α (α>0.70) [16, 17, 21, 23-25]. The Lysholm score had a lower Cronbach’s α value (0.72) as compared to the other instruments that were tested for internal consistency [16]. The rest of the measures lacked evidence for internal consistency [18, 20, 22, 23]. The majority of the measures were evaluated for test-retest reliability [15-17, 19, 21-23, 25, 26], with five of them having acceptable ICC values (ICC ≥ 0.70) [15-17, 22, 23, 25, 26]. The Lysholm score had the highest value (0.94) [16], and only the ACL-QOL score had a low ICC value (0.60) [19, 21].

Validity: Content validity was assessed for all the outcome measures, apart from the original ACL-RSI scale [25]. In their approach, floor and ceiling effects (FCEs) were used as measures of content validity to show that the instrument had a full range of available scores and was acceptable for all the reported outcome measures. Criterion validity was assessed in some of the measures [16-18, 20, 23, 24, 26], and IKDC was used as the “gold standard” in the majority of the studies showing good correlation.

Responsiveness: Effect sizes and standardized response means of measures from patients with reported improvement in scores were reported for only four measures [15, 16, 19, 21-23]. For three measures (Lysholm score, TAS, and Cincinnati score), ES and SRMs were large (≥0.80), and for one measure (ACL-QOL), ES was moderate.

Risk of Bias Assessment

When assessing the risk of bias for included studies, the RCT had unclear risk of bias [11], with unclear methods for generating and concealing the allocation sequence, and no clear measures about blinding study participants and personnel [22]. For prospective cohort studies, the risk of bias assessment is presented in Table 4. The majority of the studies were of high quality, scoring high in the assessment [15, 16, 18, 20, 21, 25, 26], with four of them scoring the highest score of nine stars [15, 18, 25, 26]. Two studies were of fair quality, scoring seven stars in the assessment [17, 24]. A risk of bias assessment for the two case-series studies was not performed, as such studies are considered low-quality by design [19, 23].

**Table 4:** Risk of bias assessment for prospective cohort studies using the Newcastle-Ottawa Scale (NOS) A study can be awarded a maximum of one star for each question and a maximum of two stars for the comparability of cohorts. The more stars a study is awarded, the lower the risk of bias. The threshold for “good quality”: three or four stars in the selection domain, one or two stars in the comparability domain, and two or three stars in the outcome/exposure domain. The asterisks represent stars. [12]

Lead author (year)	Representativeness of cohort	Selection of non-exposed cohort	Ascertainment of exposure	Demonstration of that outcome was not present at the start of the study	Comparability of cohorts	Assessment of outcome	Follow up long enough for outcomes to occur	Adequate follow-up of cohorts	NOS score
Barber-Westin et al., (1999) [15]	Somewhat representative*	Drawn from the same community as the exposed cohort*	Structured interview*	Yes*	Study controls for age, sex * Study controls for other factors: diagnosis, surgery*	Independent blind assessment*	Yes*	Complete follow-up, all subjects accounted for*	9*
Briggs et al., (2009) [16]	Truly representative*	Drawn from the same community as the exposed cohort*	Secure record*	Yes*	Study controls for age, sex*	Record linkage*	Yes*	Complete follow-up, all subjects accounted for*	8*
Irrgang et al., (2001) [17]	Somewhat representative*	Drawn from the same community as the exposed cohort*	Structured interview*	Yes*	Study controls for age, sex * Study controls for other factors: diagnosis*	Record linkage*	No	No statement	7*
Jacobs et al., (2018) [18]	Truly representative*	Drawn from the same community as the exposed cohort*	Structured interview*	Yes*	Study controls for age, sex * Study controls for other factors: race*	Record linkage*	Yes*	Subjects lost to follow-up unlikely to introduce bias: a small number lost (6%) *	9*
Marmura et al., (2023) [20]	Truly representative*	Drawn from the same community as the exposed cohort*	Structured interview*	Yes*	Study controls for age, sex*	Record linkage*	Yes*	Subjects lost to follow-up unlikely to introduce bias: a small number lost (2%)*	8*
Mohtadi et al., (1998) [21]	Truly representative*	Drawn from the same community as the exposed cohort*	Structured interview*	Yes*	Study controls for age, sex * Study controls for other factors: meniscal tear, surgery*	Independent blind assessment*	Yes*	No statement	8*
Webster et al., (2018) [24]	Truly representative*	Drawn from the same community as the exposed cohort *	Structured interview *	Yes *	Study controls for age, sex *	No description	Yes *	Complete follow-up: all subjects accounted for *	7 *
Webster et al., (2008) [25]	Truly representative*	Drawn from the same community as the exposed cohort*	Structured interview*	Yes*	Study controls for age, sex * Study controls for other factors: sport participation*	Independent blind assessment *	Yes*	Complete follow-up: all subjects accounted for*	9*
Webster et al., (2022) [26]	Truly representative*	Drawn from the same community as the exposed cohort*	Secure record *	Yes*	Study controls for age, sex * Study controls for other factors: level of sport participation*	Record linkage*	Yes*	Complete follow-up: all subjects accounted for *	9*

Discussion

This review summarized all the knee-specific outcome measures for ACL-injured knees, reporting on their content and psychometric properties, and showed that among the many knee-related scores and scales in the literature, there are eight ACL-injury-specific: the Lysholm score, the TAS, the Cincinnati score, the ACL-QOL score, the IKDC SKF, the KOOS global (short form), the KOOS-ACL score, and the ACL-RSI scale (extended and short version). All these measures were validated and tested for ACL-injured patients, but there is a variation in their reported psychometric properties. Moreover, these measures assess different aspects of knee-related health with different approaches, and this makes it very difficult to recommend only one as a PROM for ACL injury patients.

There is no standardized knee instrument/outcome measure; hence, the assessment of such a tool’s utility is based mainly on its psychometric properties and how applicable it is to the specific disease/condition. The most important factor in deciding which outcome measure to use is the evidence for its psychometric properties. These properties/parameters are closely related to the methodological quality of the tool and the quality of the information that can be obtained [8]. Good reliability means that the data are stable and consistent over time and in different contexts [6]. Validity is about ensuring that a test measures the outcome it was designed to measure, and this is evaluated mainly by estimating the extent to which a measure agrees with a “gold standard” (criterion validity”) [6]. Moreover, an instrument/tool with good responsiveness means that it is able to detect and measure changes over time in the construct to be measured [7].

Evaluation of the psychometric properties of an outcome measure is based on available evidence from testing the measure in the population of interest. The majority of the reported measures in our study were reliable with acceptable internal consistency and test-retest reliability, although there was a lack of evidence for three measures. Good evidence for reliability exists and is reported for Lysholm score and TAS [16, 23], ACL-QOL [19, 21], IKDC SKF [17, 26], and ACL-RSI [25]. Evidence for reliability is weaker for the Cincinnati score [15, 22] and KOOS-ACL [20], while there is not enough evidence for the reliability of the KOOS global (short form) [18]. Good evidence for validity is reported for Lysholm score and TAS [16, 23], IKDC SKF [17, 26], KOOS global (short form) [18], and KOOS-ACL [20]. There is not enough evidence for the validity of the Cincinnati score [15, 22], the ACL-QOL [19, 21], and the ACL-RSI [25]. Testing criterion validity, IKDC was used as the “gold standard” in all tested measures, and there was a good correlation. All studies used FCEs as measures of content validity, and they were acceptable. However, FCE is not a direct measure of content validity and is more indicative of the instrument’s potential to represent the full range of available scores and responsiveness. Only four outcome measures (Lysholm score, TAS, Cincinnati score, and ACL-QOL) have actual evidence for responsiveness to change [15, 16, 19, 21-23]. Good responsiveness with moderate to large ES and SRM is reported for all four.

Although there was a small overlap in some knee-related health dimensions (such as symptoms, pain locking, function, and sports) among some of the measures, there was a significant variation in the aspects of knee-related health that all these instruments could measure. First, the Lysholm score evaluated eight domains, including limb, support, locking, instability, pain, swelling, stair climbing, and squatting [23]. Some of these domains are measured with the Cincinnati score as well (pain, locking), along with work activity, exercise, and follow-up progress [22]. The KOOS global (short form) evaluates pain, stiffness, and function but also evaluates QOL [18]. The TAS evaluates only the highest level of activity in which the patient can participate [23]. The KOOS-ACL score measures two more generic domains of function and sports [20], the same as the IKDC SKF, which measures knee function as well [17], and the ACL-QOL, which measures symptoms and sports participation but also evaluates symptoms, work concerns, lifestyle, and social and emotional status [21]. Last, the ACL-RSI scale measures different aspects of health that have to do with emotions, confidence in performance, and risk appraisal [24, 25].

With a few instruments being tested thoroughly and having satisfactory values of reliability, validity, and responsiveness, it becomes a challenge to choose one appropriate and reliable measure for a study. When looking for a PROM, researchers and clinicians should choose among instruments with robust psychometric properties and should consider the characteristics of the patient population in which the instrument has been tested. In our review, all measures presented have been designed and tested in patients with ACL injuries, and most of them have good or acceptable psychometric properties. It is vital to have an instrument that can guide an objective comparison of outcomes following different management strategies for ACL injuries. Nevertheless, a single universal instrument for patients with ACL injuries is difficult to choose and remains a challenge. A recommendation of a group of appropriate outcome measures rather than one single measure can be made based on this review, and the researcher can choose and make their assessment.

The study has its limitations. First, this study only looked into the English version of scores, addressing only English-speaking populations. A lot more work would have been done if versions of these instruments in other languages were included, with possibly different results and conclusions regarding the reliability and validity of these measures. Consequently, results from our study can only be considered when English versions of these measures are used. Furthermore, there was an overlap in some knee-related health dimensions among the measures examined, and even these dimensions were assessed with different approaches. Last, none of the studies presenting and evaluating all these measures measured the observer reliability of these instruments, which is the ability of a single (intra-observer) or multiple observers (inter-observer) to produce the same measurements consistently under the same conditions for the same sample [31]. Measurement of this aspect of reliability would have added a lot of credit to the reliability of these measures.

## Conclusions

In conclusion, several ACL-injury-specific PROMs have been described, such as the Lysholm score, the TAS, the Cincinnati score, the ACL-QOL score, the IKDC SKF, the KOOS global (short form), the KOOS-ACL score, and the ACL-RSI scale. They all have had acceptable testing but with stronger evidence and a more robust evaluation for Lysholm score, TAS, ACL-QOL, and IKDC SKF. However, there is variation in their psychometric properties as well as the aspect of knee-related health they are assessing. Hence, none can be universally applicable to all patients with ACL injuries, and researchers need to choose a group of appropriate outcome measures rather than one single measure. Recognizing these parameters is vital when choosing which instrument to use in reporting the outcomes of ACL injury or ACL surgery studies.
